# Cooperative Evolutionary Strategy between the Bacteriome and Mycobiome

**DOI:** 10.1128/mBio.01951-16

**Published:** 2016-11-15

**Authors:** Mahmoud Ghannoum

**Affiliations:** Center for Medical Mycology, University Hospitals Cleveland Medical Center and Case Western Reserve University, Cleveland, Ohio, USA

## Abstract

Nonhealing chronic wounds are all unique in origin and circumstance, and attempting to isolate a single etiology for the failure of a wound to heal is daunting. Wounds represent complex systems of multispecies fungal and bacterial biofilms. The survival strategies of interactive microbial communities have led to cooperative evolutionary strategies that culminate in biofilm formation. In microbial dysbiosis, biofilms are beneficial to both bacterial and fungal communities but detrimental to the host. Fungi benefit by a surge in their virulence factors, while bacteria become tolerant to antibacterials as a consequence of living under the protective umbrella of the biofilm matrix. This interkingdom cooperation negatively impacts the host, as the fungi and bacteria produce extracellular enzymes that inflict tissue damage, leading to an increase in proinflammatory cytokines, which results in oxidative damage and apoptotic cell death.

## COMMENTARY

Chronic wounds are the most financially burdensome skin disease, affecting nearly 6.5 million patients in the United States, with an annual expenditure of nearly $25 billion for treatment alone (1). Additionally, the prevalence of chronic wounds is on the rise, driven by an aging population and growing incidence of diabetes mellitus and obesity worldwide (2). Furthermore, infections of combat wounds are also on the increase (3, 4). Microbial infections are recognized as one of the many destructive processes that delay wound healing. Conventional diagnostic cultures of wounds are largely biased toward microbes that are able to grow rapidly in standard culture media and are presumed to be significant. Lack of reliable diagnostic measures for wound infections leads to nontargeted use of antimicrobials, promoting development of resistant microbial strains and/or killing of potentially beneficial commensal bacteria. Therefore, further understanding of the complex relationship between microbes and delayed healing is critical for development of more evidence-based treatment strategies.

Recent studies investigating the contribution of the microbial communities to health and disease are starting to show that bacteria, fungi, and viruses contribute to our health status (5). Early glimpses at ways the mycobiome might play a positive role in our bodies have been achieved despite the many technical challenges (5). More exciting and as shown in the paper by Kalan et al. (6) and work by our group (7) is the realization that not only do bacteria and fungi coexist in different body sites, but they also interact and have evolved to cooperate in a way that is beneficial to their existence and detrimental, in some cases, to the host. This cooperation represents evolutionary strategies aimed at protecting themselves from the host and antimicrobial insults.

In their article, Kalan et al., unlike earlier studies which focused on bacteria (8, 9), longitudinally profiled 100 nonhealing diabetic foot ulcers using high-throughput sequencing and showed that up to 80% of wounds contain fungi. In contrast, cultures performed in parallel captured only 5% of colonized wounds. The findings that fungi exist in the vast majority of wounds are important since wound infections are currently considered to be bacterial in nature. This perception could be due to the fact that culture-based diagnostic approaches are largely biased toward microbes that are able to grow rapidly in standard culture media and are presumed to be significant.

In total, 17 fungal phylotypes were identified (with a relative abundance of >1%) belonging to the phylum *Ascomycota* or *Basidiomycota*, with *Cladosporium herbarum*, a known environmental fungus associated with allergy (present in 41% of the samples and 56% of subjects), followed by the pathogen *Candida albicans* (22% of samples and 47% of subjects) as the two most abundant species. Analysis of the effect of antibiotic use on microbial diversity showed that subjects who received antibiotics had significantly higher Shannon diversity indices (for all visits combined) than those subjects who did not receive an antibiotic (*P* = 0.029). However, diversity over time did not significantly fluctuate before, during, or after antibiotic administration.

A very significant finding of the study by Kalan et al. (6) is the discovery that the mycobiome was associated with clinical outcomes. Specifically, mean proportions of fungal pathogens (and not fungi associated with allergens) were higher in nonhealing wounds and those that ultimately resulted in amputation. This association extended to wound necrosis, which was distinctly associated with pathogenic fungal species and not allergenic molds. Analysis of the fungal community at baseline visit (where specimens were collected over viable wound tissue, not necrotic tissue at the initial clinical presentation and before the wound was surgically debrided of dead tissue and/or biofilms) and subsequent visits showed that the fungal distribution was only significant at the initial presentation. This suggests that the mycobiome at the presentation visit may have utility as a diagnostic marker of time to heal, as well as act as an indicator of poor prognosis (necrosis and amputation). If confirmed in future studies, this discovery may address a glaring gap in wound management, namely, the lack of reliable diagnostic markers. Therefore, further understanding of the complex relationship between microbes and delayed healing is critical for development of more evidence-based treatment strategies.

Chronic microbial infections in the form of biofilms are increasingly recognized as a common cause of delayed healing through various mechanisms (10). Biofilms are microbes embedded in a polymeric matrix that protects them from antimicrobials and resists host defenses. The study of biofilms has introduced a new paradigm of chronic microbial infections: instead of free-floating (planktonic) microbes causing disease patterns that can be reproduced following Koch’s postulates, biofilms are attached polymicrobial communities in which relationships between microbes can alter disease outcome. The use of molecular diagnostic techniques applied to biofilms in chronic wounds has shown many microbes to be in a “viable but nonculturable” state, highlighting the limitations of conventional culture techniques for understanding the composition of biofilms (11).

In the study by Kalan et al. (6), mixed-species biofilms (*C. albicans* and *Citrobacter freundii* or *Trichosporon asahii* and *Staphylococcus simulans*) formed rapidly *in vitro* and revealed close interactions between bacterial and fungal cells, with yeast cells forming the biofilm “core” and bacteria associating with the biofilm periphery, coating yeast cells and hyphae as they rapidly grew out of the agar surface (within 48 h). This observation of cooperative mixed biofilms agrees with the findings described in our recent study (7) where we discovered significant intra- as well as interkingdom associations in the bacteriome and mycobiome of Crohn’s disease (CD) patients. In our study, *C. tropicalis* exhibited significant positive association with *Serratia marcescens* and *Escherichia coli*. Similar interkingdom associations between bacterial and fungal communities in CD were recently reported by Sokol et al. (12), who showed that fungal genera (mostly *Saccharomyces* and *Malassezia*) were positively correlated with several bacterial taxa in CD.

Furthermore, like Kalan et al. (6), our *in vitro* studies showed that *C. tropicalis*, *E. coli*, and *S. marcescens* cooperate to form robust biofilms comprising fungal hyphae (7). Biofilms render the organisms resistant to antimicrobial agents and protect them from immune cells (13–15). Moreover, fungal filamentation is a known *Candida* virulence factor that damages host tissues and triggers specific host immune responses (16–19). Distinct interspecies interactions in this biofilm environment were clearly evident, where *E. coli* tended to be closely associated with the fungal cell walls, while *S. marcescens* used its fimbriae to form a “bridge” between *C. tropicalis* and *E. coli* that stabilized the bacterium-fungus biofilm structure. Interestingly, Castro et al. (20) described analogous interactions between *S. marcescens* and *Trypanosoma cruzi* cells that were mediated by d-mannose-recognizing pili in insect guts. These reports show that interkingdom interactions have evolved in mixed biofilms in many varied niches.

**FIG 1:**
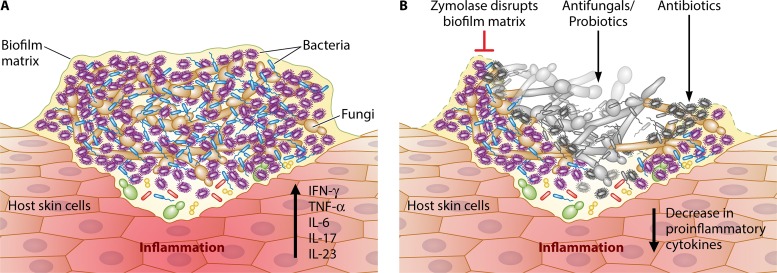
Interkingdom cooperation between fungi and bacteria. Chronic wounds are complex systems of multispecies fungal and bacterial biofilms. These biofilms provide a protected milieu for microbes living in close proximity. Fungal cells form the biofilm core while bacteria associate around the periphery of the cells. The fungal hyphae and microbial-secreted enzymes/metabolites facilitate invasion of the skin epidermis/dermis leading to host tissue damage and inflammatory response manifested by an increase in proinflammatory cytokine production (panel A). Panel B shows disruption of the biofilm matrix by zymolase thereby unmasking the microbes. Consequently, treatment with antifungal agents (e.g., echinocandins) and antibiotics leads to microbial cell death (gray/black color) and a decrease in the production of proinflammatory cytokines. IFN-γ, gamma interferon; TNF-α, tumor necrosis factor alpha; IL-6, -17, and -23, interleukins 6, 17, and 23, respectively.

One can speculate why the microbial communities have developed cooperative evolutionary strategies culminating in the development of robust thick biofilms (Fig. 1). In other words, why have fungi and bacteria evolved to adopt a biofilm lifestyle? Why would an individual species contribute to the group at the expense of its own interests? Evolutionary biologists conducted studies to understand the mechanisms sustaining the persistence of cooperation and suggested that spatial community structure (as exists in biofilms) provides a solution as to how cooperation might develop and remain stable (21). These studies provide evidence to show that within a biofilm, cooperation simultaneously results in strategies that ensure the stability of cooperative traits by directly or indirectly reducing the presence of cheaters (22). Because of its role in biofilm structure, the biofilm matrix (consisting primarily of extracellular polysaccharides [EPSs]) plays an important role in maintaining stability and cooperation within biofilms. In this regard, EPS producers play a dual role—they outcompete nonproducers in the presence of solute gradient (including the oxygen and resource gradient) and altruistically push their partners into a more nutrient-rich environment. In thick biofilms, cells far removed from the nutrient source or outside the biofilm milieu experience low levels of available nutrients, while those microbial cells living closer to the periphery will benefit from the available resources.

Such strategic benefits apply to fungal and bacterial mixed-species biofilm, as reported by Kalan et al. and Hoarau et al. (6, 7). The fungi benefit by gaining virulence factors (e.g., increased ability to form hyphae and to secrete extracellular enzymes, such as aspartic proteinase [23] relative to a monoculture biofilm), thereby enhancing their ability to invade the host. Bacteria, on the other hand, develop antibacterial tolerance afforded by living under the protective fungal matrix umbrella. Evidence to support this concept has been shown recently with *E. coli* or *Staphylococcus aureus* and *C. albicans* (24, 25). This inter- and intrakingdom cooperation impacts the host immune system, where levels of proinflammatory cytokines (e.g., Th17 cytokines) may increase under the influence of enteric pathogens and immunomodulatory components of fungal biofilms (e.g., fungal β-d-glucans and bacterial lipopolysaccharides), causing increased oxidative damage and apoptotic cell death. Additionally, microbe-induced production of mucolytic enzymes may lead to barrier dysfunction, resulting in tissue damage and lesion formation. In this regard, separate studies have shown that the bacterium *Ruminococcus gnavus* and the fungal pathogen *C. albicans* produce mucolytic enzymes that can degrade the protective mucin layer of the gut epithelium, contributing to lesion formation (26, 27).

In conclusion, observation of diverse fungal communities in chronic nonhealing wounds and their ability to form interkingdom biofilms with bacterial species emphasizes not only their paramount importance, but also the complexity of studying whole microbial communities, their interspecies interactions, and implications in chronic disease. The finding that the mycobiome is associated with clinical outcomes raises the possibility that this community may have potential clinical utility as a diagnostic and prognostic biomarker.
